# Editorial: Break the mental health stigma: PTSD

**DOI:** 10.3389/fpsyt.2024.1493657

**Published:** 2024-10-09

**Authors:** Georgina Warner, Farah Chamaa

**Affiliations:** ^1^ Child Health and Parenting (CHAP), Department of Public Health and Caring Science, Uppsala University, Uppsala, Sweden; ^2^ Biological and Environmental Sciences and Engineering Division, King Abdullah University of Science and Technology (KAUST), Thuwal, Saudi Arabia

**Keywords:** PTSD - posttraumatic stress disorder, mental health stigma, social -awareness, trauma-informed practice, trauma-specific programs

Post-traumatic stress disorder (PTSD) stands out among mental health disorders due to its clear and identifiable trigger—a traumatic event (1). This distinct characteristic might suggest that PTSD should be less stigmatized, as the cause is an external rather than an internal event. However, individuals with PTSD frequently encounter stigma and barriers to seeking care (2). This persistent stigma remains a major obstacle to effective treatment and support for those affected by PTSD. The disorder is characterized by enduring distressing symptoms following exposure to traumatic events and manifests differently across various populations and contexts.

Trauma-informed clinical practices are essential for creating a healthcare environment that is safe, supportive, and effective for all patients, especially those with a history of trauma (3). By recognizing the pervasive impact of trauma and integrating principles of safety, trust, and empowerment into care, healthcare providers can improve patient outcomes, reduce stigma, and foster a more compassionate and responsive healthcare system. Essential components of trauma-informed clinical practices include screening for trauma, training staff in trauma-specific treatment approaches, involving patients in the treatment process, and engaging referral sources and partnering organizations (3). Scientific research underpins these practices by forming an evidence base for targeted screening, training, and intervention strategies.

The articles in this Research Topic cover a diverse range of subjects: from the impact of childbirth experiences on maternal mental health to the role of interoceptive awareness in emotional regulation among PTSD patients. They also investigate the psychological toll of pandemics on healthcare workers, the efficacy of intensive treatment programs for severe PTSD, and the challenges faced by soldiers dealing with moral injury. Each study provides unique insights into the mechanisms, risk factors, and potential interventions for PTSD, highlighting its multifaceted nature and profound implications for individuals, families, and communities.

Effective PTSD care requires awareness of at-risk groups. Soldiers and military veterans are among the most commonly studied populations for PTSD. In this Research Topic, Diekmann et al. examined the effectiveness of value-based cognitive-behavioral group therapy in addressing war-related moral injury. Their research integrates cognitive-behavioral and spiritual care approaches, offering new insights into treating moral injury and highlighting the importance of addressing moral and ethical dimensions in trauma therapy. Another at-risk group highlighted by the Research Topic is women experiencing childbirth and developing postpartum PTSD. Gilbert et al. found that assessing maternal and infant life threat shortly after childbirth significantly influences maternal mental health outcomes, underscoring the need for targeted screening and early support interventions in the postpartum period. Handelzalts et al. explored how postpartum social support and relationship quality can alleviate symptoms of childbirth-related PTSD. Both papers suggest translational impacts, with the former providing insights for routine childbirth PTSD screening and the latter focusing on postpartum support interventions.

Healthcare providers themselves are also at risk, often facing distressing experiences that can lead to PTSD (1), previously referred to as Secondary Traumatic Stress. Zhang et al. investigated the prevalence and influencing factors of PTSD among Chinese healthcare workers during the COVID-19 pandemic. Their comprehensive meta-analysis identified risk and protective factors, such as increased risk associated with being female, having fewer years of experience, and working on the frontline, while social support and psychological resilience emerged as key protective factors. These findings underscore the urgent need for targeted psychological interventions and support systems for healthcare workers, especially those with identified risk factors, to mitigate the long-term mental health impacts of the pandemic.

Not everyone who experiences trauma develops PTSD. The onset of the condition is influenced by various factors including previous trauma and pre-existing mental health conditions (4, 5). However, post-trauma factors seem more predictive (4, 5). Individual emotional responses to trauma and social support are significant influencing factors (4, 5), with family functioning being particularly important for children (4). These observations are echoed in the papers within this Research Topic. Handelzalts et al. highlight social support in the context of childbirth PTSD, while Zhang et al. emphasize its importance for healthcare workers. Yuan et al. examined trauma among children and adolescents in northern China, highlighting significant variations in PTSD prevalence based on demographic factors such as age, living environment, and parental marital status. These findings highlight the necessity for targeted psychological interventions and support systems tailored to the unique needs of traumatized youth.

An important aspect of PTSD care is treatment tolerability, which impacts patient adherence and the overall success of treatment. Dropout rates from recommended psychological therapies for PTSD are high, particularly for trauma-focused interventions (6). Gahnfelt et al.‘s evaluation of an 8-day intensive treatment for severe PTSD suggests that while intensive treatments can be beneficial, they also present challenges. Their study reported a low rate of treatment dropout, reduced PTSD symptom burden, and no adverse effects, indicating that intensive treatment is a viable option, though further research is needed to confirm its effectiveness.

Addressing PTSD comprehensively involves understanding and treating both its psychological and physiological aspects. Individuals with PTSD often experience heightened arousal symptoms, such as increased heart rate, hypervigilance, and exaggerated startle responses (1). In their paper, Leech et al. explored the relationship between PTSD and interoceptive awareness - the ability to perceive internal bodily sensations. Chronic stress associated with PTSD can dysregulate the body’s stress response system, leading to long-term neurobiological and hormonal changes (7), which may heighten the risk of physical health conditions. Enhancing interoceptive awareness could improve emotional resilience and reduce PTSD symptom severity. Within this Research Topic, Jiang and Yang investigated the association between PTSD and cancer risk, finding limited evidence suggesting a link between PTSD ovarian cancer. This underlines the need for further research into the biological mechanisms underlying such associations.

In summary, the collection of papers within this Research Topic highlights the need for a comprehensive understanding of PTSD and trauma-informed clinical practice. The findings collectively advocate for a more nuanced and compassionate approach to PTSD care, emphasizing the integration of psychological, social, and biological perspectives in designing tailored interventions. This integration allows clinicians to develop personalized treatment plans that are more effective and holistic. Such an approach facilitates early identification, appropriate intervention, and continuous support, leading to improved symptom management, enhanced quality of life, and reduced risk of comorbid conditions. By increasing awareness and understanding of PTSD across healthcare settings, educational institutions, and communities, we can destigmatize mental health issues and promote early detection and intervention. Moreover, continued investment in research is crucial for advancing our knowledge of PTSD mechanisms and treatment outcomes, paving the way for innovative therapeutic approaches and evidence-based practices. Moving forward, collaborative research and interdisciplinary partnerships are essential for translating knowledge into actionable strategies that promote resilience, recovery, and well-being. By fostering a culture of empathy, inclusivity, and scientific rigor, we can create a future where PTSD is addressed with understanding, compassion, and effective treatment options.
